# Dimensional Accuracy of Stone Casts Obtained with Multiple Pours into the Same Mold

**DOI:** 10.5402/2012/730674

**Published:** 2012-12-24

**Authors:** Valdimar da Silva Valente, Artêmio Luiz Zanetti, Pedro Paulo Feltrin, Ricardo Tatsuo Inoue, Carmem Dolores Vilarinho Soares de Moura, Luiz Evaldo de Moura Pádua

**Affiliations:** ^1^Department of Restorative Dentistry, Federal University of Piaui (UFPI), Terezina, PI, Brazil; ^2^Department of Removable Prosthodontics, University of São Paulo City, São Paulo, SP, Brazil; ^3^MSc Program in Dental Prosthesis, School of Dentistry, São Leopoldo Mandic Dental Research Center, Campinas, SP, Brazil; ^4^MSc Program in Dental Prosthesis, São Leopoldo Mandic Dental Research Center, Campinas, SP, Brazil; ^5^Department of Plant Science, Federal University of Piaui (UFPI), Terezina, PI, Brazil

## Abstract

*Aim*. The aim of the present study was to compare the dimensional accuracy of stone casts obtained with vinyl polysiloxane molds through the double-impression technique with three pours into the same mold. *Methods*. A stainless steel master model was constructed simulating a three-unit fixed prosthesis. Twelve impressions were taken of this master model with addition silicone, using the double-impression technique. Three pours of type IV gypsum were then made into each mold, thus producing 36 casts. The pours were made 1 hour, 6 hours and 24 hours after the impression procedure. Next, intra- and interabutment measurements were made in a coordinate measuring machine. *Results*. Comparative analysis of the dimensional accuracy of stone casts resulting from multiple pours was not statistically significant in pours first and second (*P* > 0.05). These values, however, were statistically significant at third pour in the height in abutment 1 and upper distance interabutment. *Conclusion*. The wait time (1 hour, and 6 hours) observed before pouring the stone into the same molds did not cause significant dimensional accuracy of the casts.

## 1. Introduction

In order to construct a fixed prosthesis, a stone die must be made by cutting the stone cast that was obtained through an impression technique. Separated from the cast, this die enables improved marginal adaptation of the prosthetic crown that will be constructed on it. Although current techniques for making removable stone dies have developed and become increasingly more accurate, the cutting out of a stone die results in significant dimensional change in the distances between abutments [1].

In this scenario, producing more than one cast from the same mold may be an option for preserving the marginal adaptation of prostheses—a result for which obtaining stone dies is required—while at the same time, preserving the dimensional accuracy of the distances between the prosthetic abutments [2].

A deficiency to making impressions in fixed prosthodontics is failure to follow basic principles inherent to the manipulation of impression materials. Stock trays are used extensively, and the importance of control of bulk is ignored. Putty/wash materials also are used extensively, usually in an inappropriate manner, resulting in impressions with less than optimal accuracy [3]. Since the most costly item of any oral rehabilitation treatment is the clinical time of the dental professional, the possibility of obtaining several casts from the same mold without changing their characteristics and dimensions could contribute to reducing the professional's clinical time, therefore reducing the overall cost of the prosthesis.

The aim of the present study was to compare the dimensional accuracy of stone casts obtained with vinyl polysiloxanemolds through the double-impression technique with three pours into the same mold.

## 2. Methods

**Figure 1:**
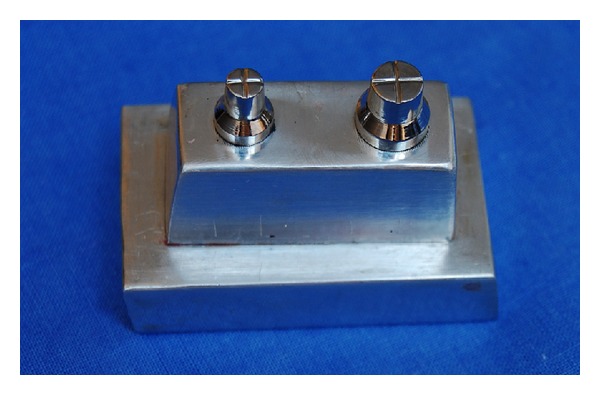
Master model.

**Figure 2:**
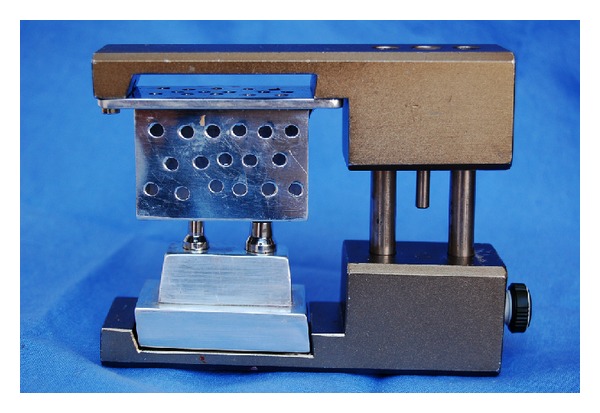
Master model with custom tray in a customized device, simulating a three-unit fixed prosthesis.

To carry out this experiment, a stainless steel master model was constructed to simulate a fixed partial prosthesis with one pontic and two crowns. At its base, the first abutment was 7 mm in height by 5 mm in diameter, while the second abutment was 7 mm in height by 7 mm in diameter. The abutments were placed 11 mm apart (Figure 1). Twelve individual rectangular perforated trays by aluminum were constructed to hold the impression material. The trays were 2.5 cm wide, 5 cm long, and 3.5 cm high, on a base 3 cm wide by 7 cm long. Subsequently, each perforated tray was properly positioned and immobilized in the upper part of a verticulator (Bio Art-São Carlos, SP, Brazil) to allow only vertical movement, thus standardizing the impression procedure by ruling out any interference by the operator (Figure 2). The master model was then centralized and fixed to the lower part of the verticulator by means of two fixation screws [4].

Twelve impressions were taken from the master model using the double-molding technique that consists of two consecutive procedures: the first is performed with the putty phase (Elite H-D+ putty soft, Zhermack, Italy, lot 38249) of the impression material and the second with the light-body phase (Elite H-D+ light body, Zhermack, Italy, lot 43096), by showing similar studies of comparable sample size [4]. A digital precision scale was used to weigh 15 g of base mass and 15 g of catalyst mass of the vinyl polysiloxane (Elite H-D+, Zhermack, Italy,) impression material used in this study, following the manufacturer's instructions. A uniform 1.0 mm space was created by applying a vacuum-formed resin sheet over the master model [5]. Mixing of the masses (Elite H-D+ putty soft, Zhermack, Italy, lot 38249) was manually carried out for 30 seconds. In the second step, the resin sheet spacer was removed from the mold in order to create the space that would be occupied by the light-body material (Elite H-D+ light body, Zhermack, Italy, lot 43096). This light-consistency material was then handled and inserted into the tray using the self-mixing device provided by the manufacturer. The tray was completely filled, and the impression was taken as before, with the putty-consistency phase [6]. After the impression procedure, all twelve impressions were rinsed in running water for 10 seconds. A disinfecting procedure was then carried out by immersing the impressions in a glass container with 350 mL of 2% glutaraldehyde (Cinord Nordeste, PE, Brazil) for 10 minutes [7].

As recommended by the manufacturer, 30 g of powdered gypsum was weighed using a digital scale (MX-BL01, Maxilife, BA, Brazil) and 6 mL of distilled water was measured using a pipette in order to obtain a powder/water proportion of 100 g of type IV special gypsum (Elite rock-Zhermack, Italy, lot 63977) to 20 mL of water. The mixing was initially done manually in a rubber mortar with a spatula for plaster use and afterwards in a vacuum (Turbomix-EDG, SP, Brazil) mixer for 30 s, to avoid bubbles in the models. The mixture was poured inside the mold under mechanical vibration (KVN, SP, Brazil), with the aid of a spatula until the mold was completely filled with a slight excess. An aluminum plate (3 cm × 4 cm) with retentive areas was then placed on top of the cast to facilitate the removal of the cast without damaging the mold. This set was removed from the mold one hour after mixing began. The second and third casts, obtained using the same mold, were produced by repeating the same disinfection, pouring and mold removing procedures [8].

**Figure 3:**
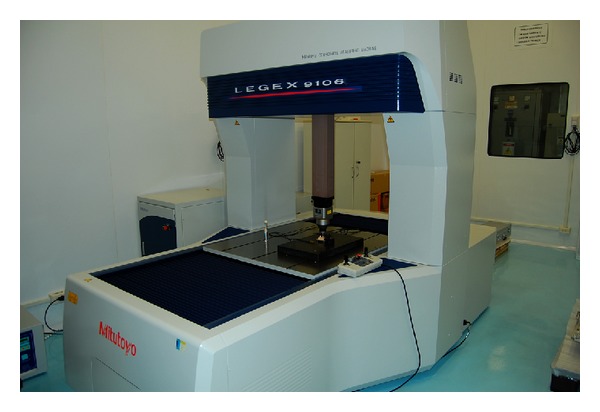
Coordinates measurement device (scanner Mitutoyo).

**Figure 4:**
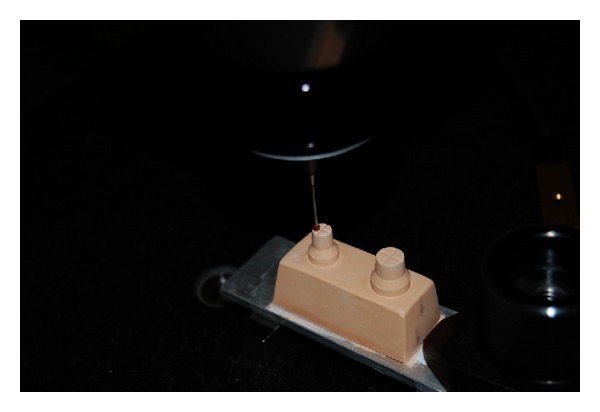
Stone cast with ruby pointer of coordinates measurement device.

All gypsum casts were measured at the metrology laboratory (Mitutoyo, Suzano-SP, Brazil). The test samples measurements were carried out using a coordinates measurement device (Model Legex 9106, Mitutoyo, Suzano-SP, Brazil) in a 20°C constant temperature environment (Figure 3), seven days after obtaining the casts, when they were completely dry. The measurements were performed through 50 contact points between a ruby pointer and the specimen (scanning). This information was then entered into a computer, coupled to the device, which produced a picture of the object (Figure 4). The digital drawing of the models, obtained from this scanning procedure, was used to calculate all the intra- and interabutment measurements of the casts and the master model. Intra-abutment measurements included lower diameter, upper diameter, and height, whereas interabutment measurements were calculated using the center of each abutment as a reference. All measurements were made with an accuracy of up to 0.01 *μ*m [4]. This scanning measurement method is more accurate than using a microscope (which requires a human operator), because the measurements are automatically calculated by computer software [4]. The measuring began with the master model, whose values were compared with those of the gypsum casts.

**Figure 5:**
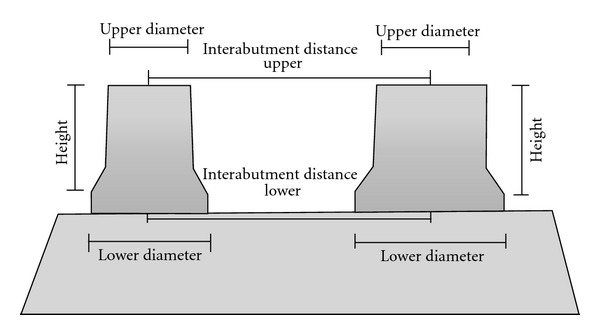
Schematic drawing of the measurements made on the models.

The abutment representing tooth 45 was called abutment 1, while that representing tooth 47 was called abutment 2. The position of the abutments was assessed by two measurements (Figure 5). Statistical analysis was performed using analysis of variance and the Tukey test at 5% probability.

## 3. Results

The results in Table 1 showed no statistically significant difference among measurements of the diameter, height, and distance interabutments of the stone casts obtained from the same mold at different times (1 hour and 6 hours). Furthermore, the values obtained from the third pour (24 hours) differed from the casts obtained in first and second pours, in height abutment 1 (*P* < 0.0001) and upper distance interabutment (*P* = 0.001) in stone casts obtained in 1 hour (1st pour); the other measurements showed no statistically significant differences. 

## 4. Discussion

In this study, casts obtained from the first pour, at one hour, the results found showed smaller variations than those shown in other studies, in which the same impression technique was used [9]. In one such study, variations were 110 *μ*m for the diameter of the abutment, 50 *μ*m for the height of the abutment, and 50 *μ*m for the distance between abutments [10].

A second cast is sometimes necessary to complete or improve the marginal adaptation when the original cast is inadvertently fractured or broken in a critical area or if a defect is noted in the cervical margin of a prepared tooth during the impression procedure [2]. The second pours were carried out 6 hours after the impression. The data show that there was no significant difference between casts of the second pour and the first pour models, indicating that it is possible to obtain more than one cast from the same mold without significant dimensional alterations. A similar result was obtained for the second pour, from the same mold, 4 hours after the impression procedure [11–13]. It was also found that repeated pours in the same mold, 24 hours and 7 days after the impression procedure, also did not affect the fidelity and dimensional stability of the gypsum casts [14].

In this study, the third pour took place 24 hours after the impression was taken. On the casts thus obtained, the results were similar to those observed on the casts obtained from the second pour for diameter abutments and lower distance interabutments. However, the height (32.08 *μ*m) and upper distance interabutment (27.77 *μ*m) measurements were higher than in the other models, unlike the measurements observed in the casts obtained from the second and third pours, with statistically significant difference (*P* < 0.05); however, these differences are very small which cannot cause clinically significant alteration; those might be attributed to the inherent properties of each material and due to the impression technique. Similar data were found when investigating the dimensional changes of gypsum casts in my study; the models produced after long periods (1, 3, and 7 days after pouring) were as accurate as those produced 10 minutes after the impression procedure [15–18].

The position of the abutments in this study was assessed through measurements of two distances between the abutments (upper and lower). The data revealed an increasing variation, with the highest variation (upper) being observed for the casts of the third pour. The measurements of the casts of the first and second pours were not statistically different from one another, but the casts from third pour were statistically different from those of the others models. Other studies have shown variations of up to 50 *μ*m in gypsum casts [14]. Changes in the distances between the abutments can compromise the marginal adaptation of fixed prostheses [14]. Values similar to those obtained in the present study have been found with variations of up to 20 *μ*m for the distance between abutments [4, 7–9]. 

Making more than one cast from the same mold is a useful procedure for optimizing subsequent laboratory processing, thus reducing the cost of a fixed partial denture. The first cast can be used for making removable die casts, which are indispensable to the marginal adaptation of the prosthetic crown. The second (in which the die cast is not cut out) can be used for adjusting proximal contact points, and the third can be used as a replacement in the event that the working cast is inadvertently fractured or a critical area of the cavity preparation, like its cervical margin, is defective because of a flawed pour of the stone into the mold. 

## 5. Conclusion

Based on the methodology used, it may be concluded that the wait time (1 hour and 6 hours) observed before pouring the stone into the same molds did not cause significant dimensional alterations of the casts. 

## Figures and Tables

**Table 1:** Mean values, standard deviation, and significant test (*P*) of the difference dimension of stone casts from master cast (*μ*m).

Measurement	First pour		Second pour		Third pour		*P* value
	Mean	Standard deviation	Mean	Standard deviation	Mean	Standard deviation	
Lower diameter (abutment 1)	21.29	18.40	19.71	15.60	19.41	16.90	0.9315
Upper diameter (abutment 1)	9.42	8.47	10.59	5.67	10.72	9.37	0.6076
Height (abutment 1)	5.02	3.28	10.85	5.71	32.08	14.55	<0.0001
Lower diameter (abutment 2)	32.85	23.16	31.29	21.38	31.04	24.61	0.9513
Upper diameter (abutment 2)	12.75	11.18	13.08	7.50	12.27	11.59	0.9495
Height (abutment 2)	20.68	18.11	17.95	14.35	25.55	13.64	0.03634
Lower distance interabutment	19.30	13.25	24.16	12.86	24.66	13.47	0.5561
Upper distance interabutment	16.23	6.49	23.99	6.71	27.77	7.07	0.001
